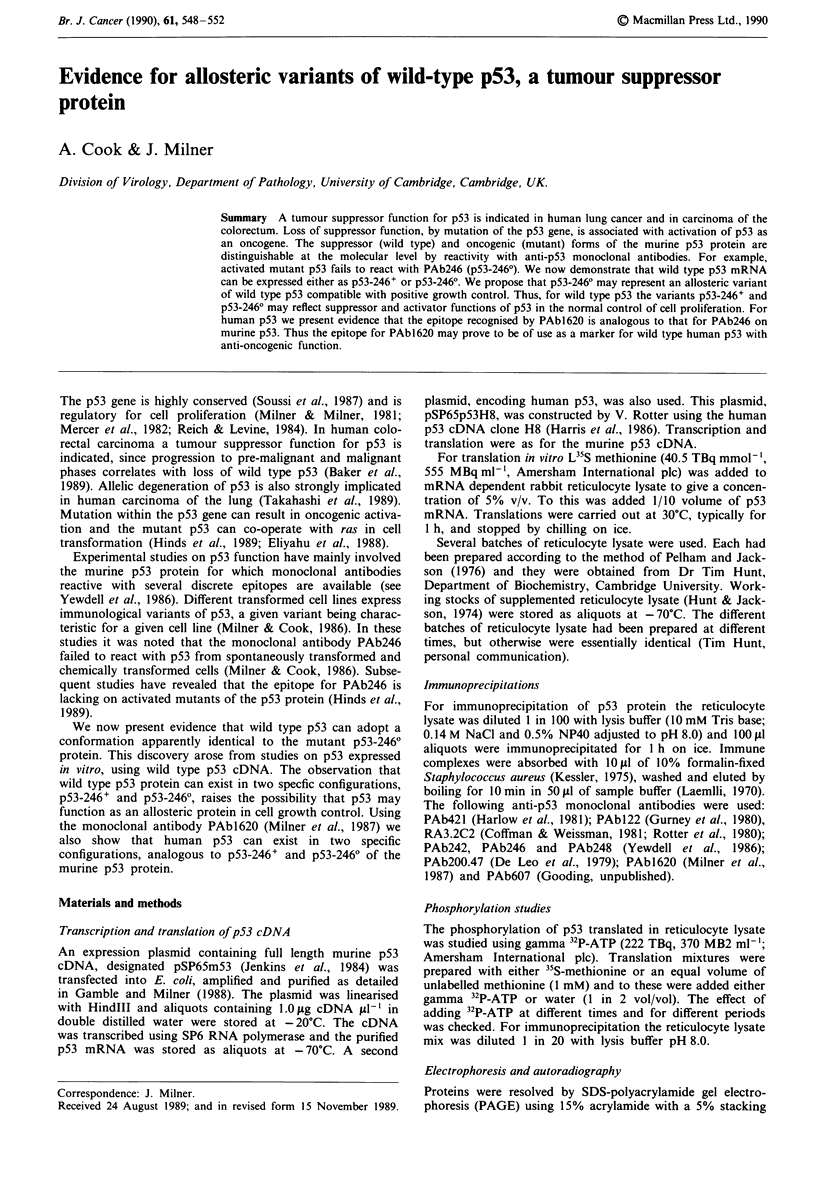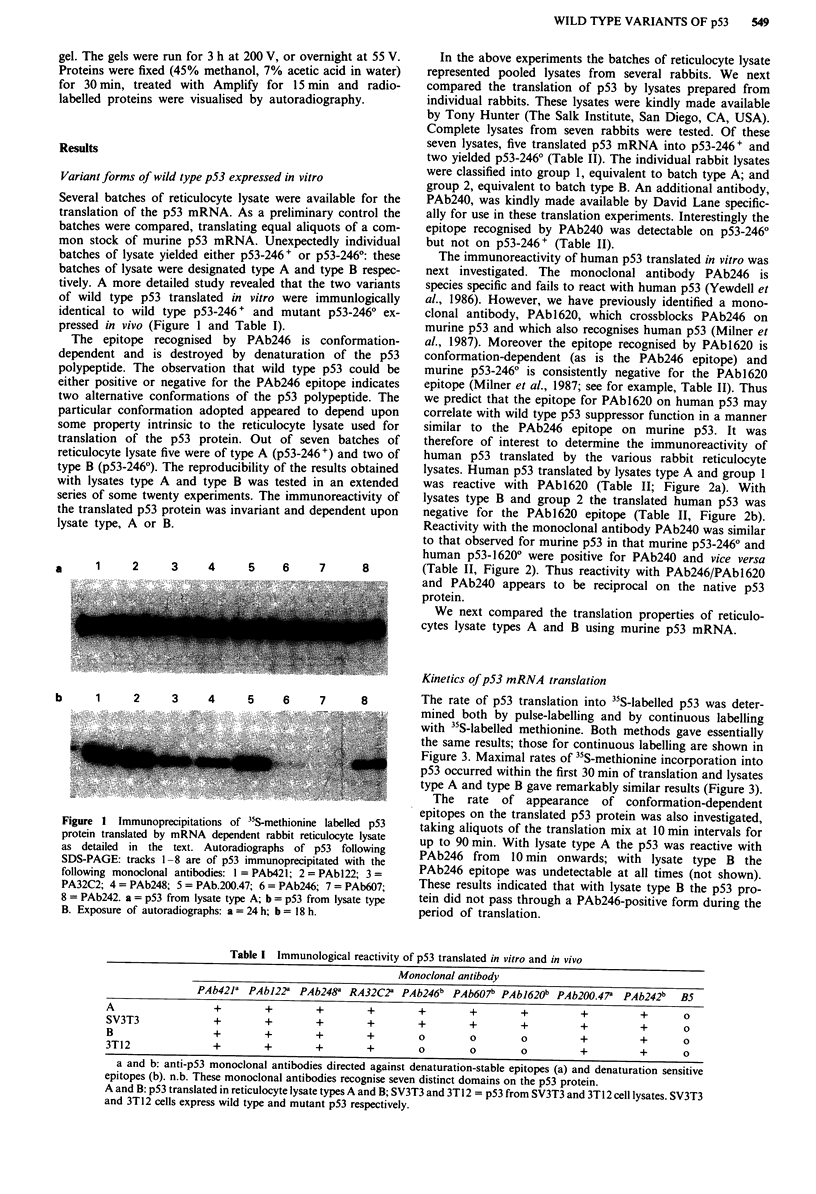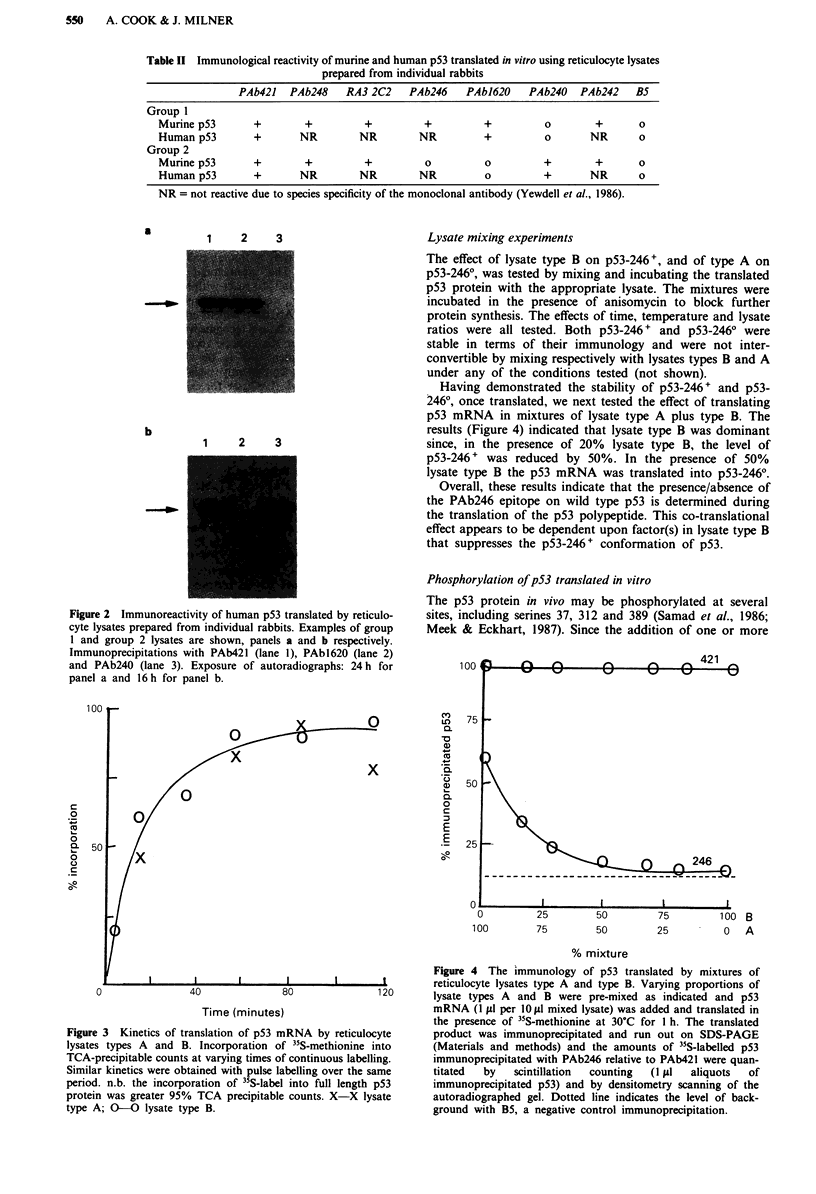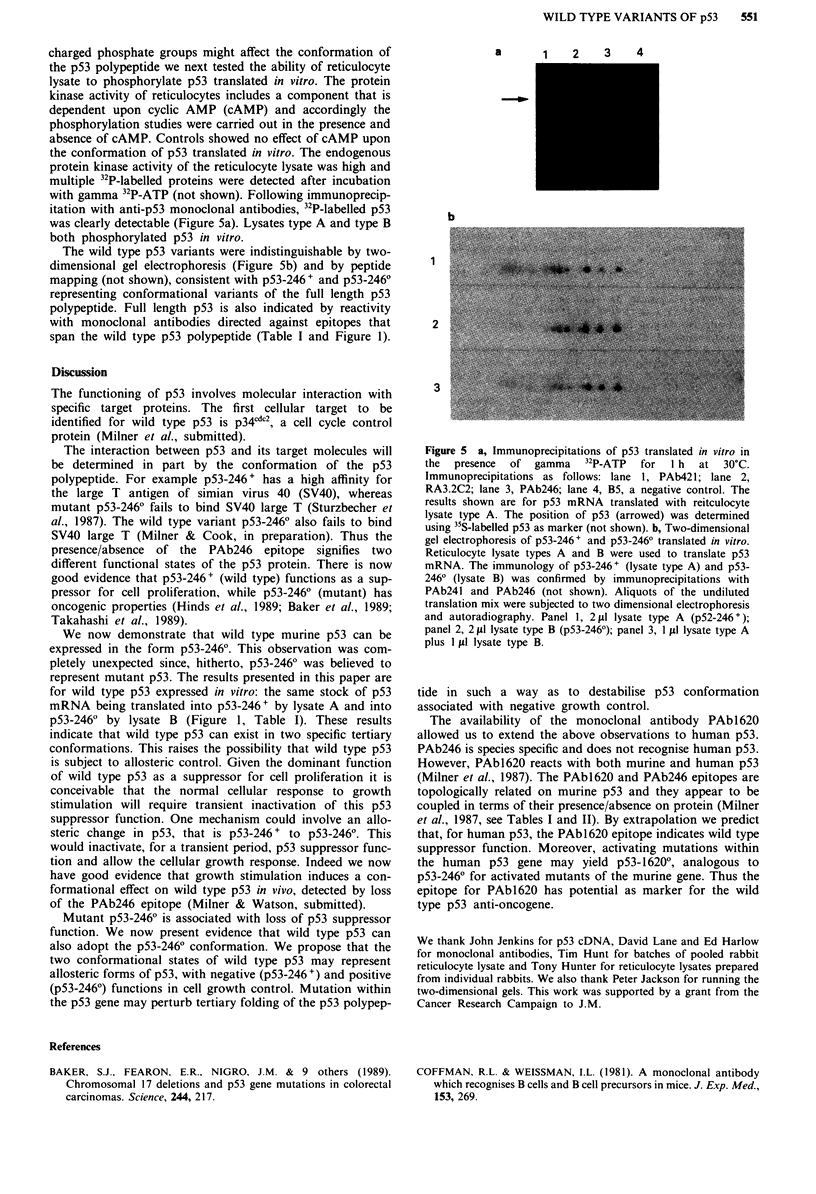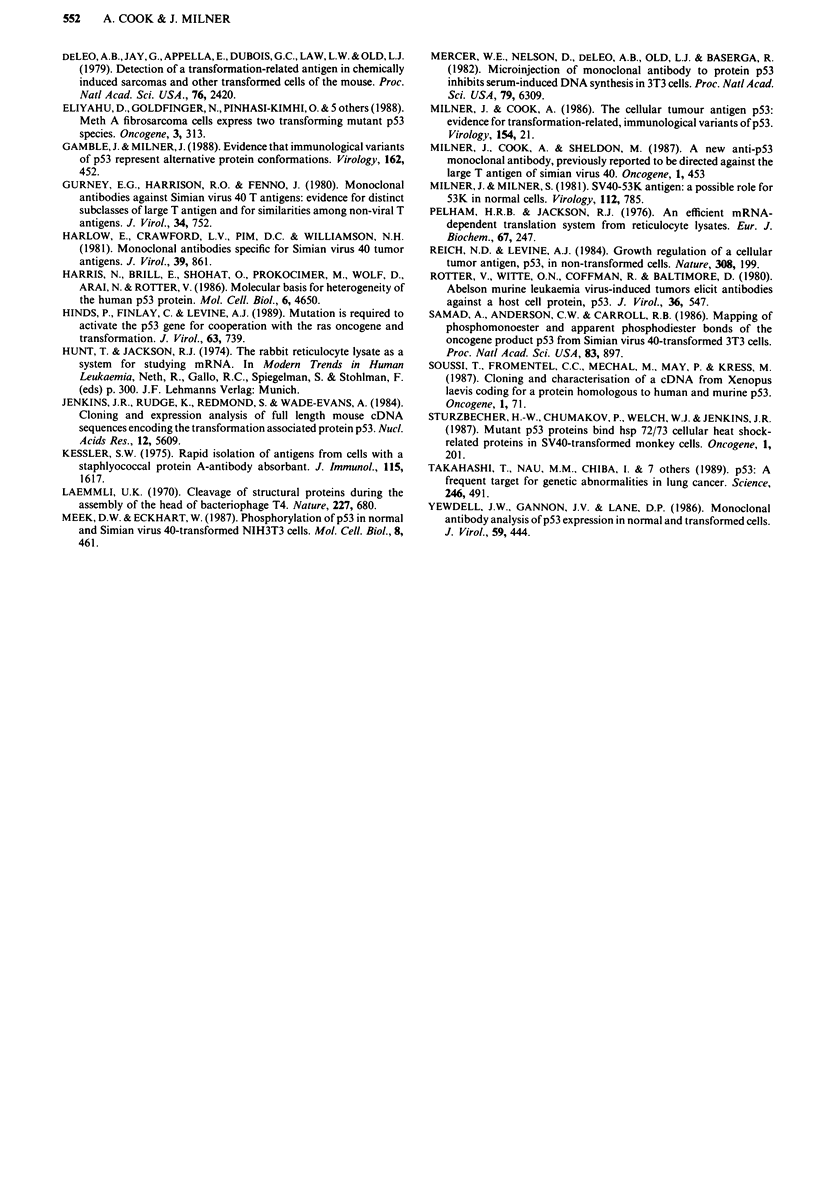# Evidence for allosteric variants of wild-type p53, a tumour suppressor protein.

**DOI:** 10.1038/bjc.1990.123

**Published:** 1990-04

**Authors:** A. Cook, J. Milner

**Affiliations:** Department of Pathology, University of Cambridge, UK.

## Abstract

**Images:**


*Br. J. Cancer* (1990), **61**, 548–552 © Macmillan Press Ltd., 1990

### Evidence for allosteric variants of wild-type p53, a tumour suppressor protein

A. Cook & J. Milner

*Division of Virology, Department of Pathology, University of Cambridge, Cambridge, UK.*

**Summary** A tumour suppressor function for p53 is indicated in human lung cancer and in carcinoma of the colorectum. Loss of suppressor function, by mutation of the p53 gene, is associated with activation of p53 as an oncogene. The suppressor (wild type) and oncogenic (mutant) forms of the murine p53 protein are distinguishable at the molecular level by reactivity with anti-p53 monoclonal antibodies. For example, activated mutant p53 fails to react with PAb246 (p53-246°). We now demonstrate that wild type p53 mRNA can be expressed either as p53-246$^+$ or p53-246°. We propose that p53-246° may represent an allosteric variant of wild type p53 compatible with positive growth control. Thus, for wild type p53 the variants p53-246$^+$ and p53-246° may reflect suppressor and activator functions of p53 in the normal control of cell proliferation. For human p53 we present evidence that the epitope recognised by PAb1620 is analogous to that for PAb246 on murine p53. Thus the epitope for PAb1620 may prove to be of use as a marker for wild type human p53 with anti-oncogenic function.

The p53 gene is highly conserved (Soussi *et al.*, 1987) and is regulatory for cell proliferation (Milner & Milner, 1981; Mercer *et al.*, 1982; Reich & Levine, 1984). In human colorectal carcinoma a tumour suppressor function for p53 is indicated, since progression to pre-malignant and malignant phases correlates with loss of wild type p53 (Baker *et al.*, 1989). Allelic degeneration of p53 is also strongly implicated in human carcinoma of the lung (Takahashi *et al.*, 1989). Mutation within the p53 gene can result in oncogenic activation and the mutant p53 can co-operate with *ras* in cell transformation (Hinds *et al.*, 1989; Eliyahu *et al.*, 1988).

Experimental studies on p53 function have mainly involved the murine p53 protein for which monoclonal antibodies reactive with several discrete epitopes are available (see Yewdell *et al.*, 1986). Different transformed cell lines express immunological variants of p53, a given variant being characteristic for a given cell line (Milner & Cook, 1986). In these studies it was noted that the monoclonal antibody PAb246 failed to react with p53 from spontaneously transformed and chemically transformed cells (Milner & Cook, 1986). Subsequent studies have revealed that the epitope for PAb246 is lacking on activated mutants of the p53 protein (Hinds *et al.*, 1989).

We now present evidence that wild type p53 can adopt a conformation apparently identical to the mutant p53-246° protein. This discovery arose from studies on p53 expressed *in vitro*, using wild type p53 cDNA. The observation that wild type p53 protein can exist in two specfic configurations, p53-246$^+$ and p53-246°, raises the possibility that p53 may function as an allosteric protein in cell growth control. Using the monoclonal antibody PAb1620 (Milner *et al.*, 1987) we also show that human p53 can exist in two specific configurations, analogous to p53-246$^+$ and p53-246° of the murine p53 protein.

### Materials and methods

### *Transcription and translation of p53 cDNA*

An expression plasmid containing full length murine p53 cDNA, designated pSP65m53 (Jenkins *et al.*, 1984) was transfected into *E. coli*, amplified and purified as detailed in Gamble and Milner (1988). The plasmid was linearised with HindIII and aliquots containing 1.0 μg cDNA $\mu l^{-1}$ in double distilled water were stored at −20°C. The cDNA was transcribed using SP6 RNA polymerase and the purified p53 mRNA was stored as aliquots at −70°C. A second plasmid, encoding human p53, was also used. This plasmid, pSP65p53H8, was constructed by V. Rotter using the human p53 cDNA clone H8 (Harris *et al.*, 1986). Transcription and translation were as for the murine p53 cDNA.

For translation *in vitro* $L^{35}S$ methionine (40.5 TBq $mmol^{-1}$, 555 MBq $ml^{-1}$, Amersham International plc) was added to mRNA dependent rabbit reticulocyte lysate to give a concentration of 5% v/v. To this was added 1/10 volume of p53 mRNA. Translations were carried out at 30°C, typically for 1 h, and stopped by chilling on ice.

Several batches of reticulocyte lysate were used. Each had been prepared according to the method of Pelham and Jackson (1976) and they were obtained from Dr Tim Hunt, Department of Biochemistry, Cambridge University. Working stocks of supplemented reticulocyte lysate (Hunt & Jackson, 1974) were stored as aliquots at −70°C. The different batches of reticulocyte lysate had been prepared at different times, but otherwise were essentially identical (Tim Hunt, personal communication).

### *Immunoprecipitations*

For immunoprecipitation of p53 protein the reticulocyte lysate was diluted 1 in 100 with lysis buffer (10 mM Tris base; 0.14 M NaCl and 0.5% NP40 adjusted to pH 8.0) and 100 μl aliquots were immunoprecipitated for 1 h on ice. Immune complexes were absorbed with 10 μl of 10% formalin-fixed *Staphylococcus aureus* (Kessler, 1975), washed and eluted by boiling for 10 min in 50 μl of sample buffer (Laemlli, 1970). The following anti-p53 monoclonal antibodies were used: PAb421 (Harlow *et al.*, 1981); PAb122 (Gurney *et al.*, 1980), RA3.2C2 (Coffman & Weissman, 1981; Rotter *et al.*, 1980); PAb242, PAb246 and PAb248 (Yewdell *et al.*, 1986); PAb200.47 (De Leo *et al.*, 1979); PAb1620 (Milner *et al.*, 1987) and PAb607 (Gooding, unpublished).

### *Phosphorylation studies*

The phosphorylation of p53 translated in reticulocyte lysate was studied using gamma $^{32}P$-ATP (222 TBq, 370 MB2 $ml^{-1}$; Amersham International plc). Translation mixtures were prepared with either $^{35}S$-methionine or an equal volume of unlabelled methionine (1 mM) and to these were added either gamma $^{32}P$-ATP or water (1 in 2 vol/vol). The effect of adding $^{32}P$-ATP at different times and for different periods was checked. For immunoprecipitation the reticulocyte lysate mix was diluted 1 in 20 with lysis buffer pH 8.0.

### *Electrophoresis and autoradiography*

Proteins were resolved by SDS-polyacrylamide gel electrophoresis (PAGE) using 15% acrylamide with a 5% stacking

Correspondence: J. Milner.
Received 24 August 1989; and in revised form 15 November 1989.

gel. The gels were run for 3 h at 200 V, or overnight at 55 V. Proteins were fixed (45% methanol, 7% acetic acid in water) for 30 min, treated with Amplify for 15 min and radiolabelled proteins were visualised by autoradiography.

### Results

### *Variant forms of wild type p53 expressed in vitro*

Several batches of reticulocyte lysate were available for the translation of the p53 mRNA. As a preliminary control the batches were compared, translating equal aliquots of a common stock of murine p53 mRNA. Unexpectedly individual batches of lysate yielded either p53-246$^+$ or p53-246$^o$: these batches of lysate were designated type A and type B respectively. A more detailed study revealed that the two variants of wild type p53 translated *in vitro* were immunlogically identical to wild type p53-246$^+$ and mutant p53-246$^o$ expressed *in vivo* (Figure 1 and Table I).

The epitope recognised by PAb246 is conformation-dependent and is destroyed by denaturation of the p53 polypeptide. The observation that wild type p53 could be either positive or negative for the PAb246 epitope indicates two alternative conformations of the p53 polypeptide. The particular conformation adopted appeared to depend upon some property intrinsic to the reticulocyte lysate used for translation of the p53 protein. Out of seven batches of reticulocyte lysate five were of type A (p53-246$^+$) and two of type B (p53-246$^o$). The reproducibility of the results obtained with lysates type A and type B was tested in an extended series of some twenty experiments. The immunoreactivity of the translated p53 protein was invariant and dependent upon lysate type, A or B.

**Figure 1** Immunoprecipitations of $^{35}$S-methionine labelled p53 protein translated by mRNA dependent rabbit reticulocyte lysate as detailed in the text. Autoradiographs of p53 following SDS-PAGE: tracks 1–8 are of p53 immunoprecipitated with the following monoclonal antibodies: 1 = PAb421; 2 = PAb122; 3 = PA32C2; 4 = PAb248; 5 = PAb.200.47; 6 = PAb246; 7 = PAb607; 8 = PAb242. **a** = p53 from lysate type A; **b** = p53 from lysate type B. Exposure of autoradiographs: **a** = 24 h; **b** = 18 h.

In the above experiments the batches of reticulocyte lysate represented pooled lysates from several rabbits. We next compared the translation of p53 by lysates prepared from individual rabbits. These lysates were kindly made available by Tony Hunter (The Salk Institute, San Diego, CA, USA). Complete lysates from seven rabbits were tested. Of these seven lysates, five translated p53 mRNA into p53-246$^+$ and two yielded p53-246$^o$ (Table II). The individual rabbit lysates were classified into group 1, equivalent to batch type A; and group 2, equivalent to batch type B. An additional antibody, PAb240, was kindly made available by David Lane specifically for use in these translation experiments. Interestingly the epitope recognised by PAb240 was detectable on p53-246$^o$ but not on p53-246$^+$ (Table II).

The immunoreactivity of human p53 translated *in vitro* was next investigated. The monoclonal antibody PAb246 is species specific and fails to react with human p53 (Yewdell *et al.*, 1986). However, we have previously identified a monoclonal antibody, PAb1620, which crossblocks PAb246 on murine p53 and which also recognises human p53 (Milner *et al.*, 1987). Moreover the epitope recognised by PAb1620 is conformation-dependent (as is the PAb246 epitope) and murine p53-246$^o$ is consistently negative for the PAb1620 epitope (Milner *et al.*, 1987; see for example, Table II). Thus we predict that the epitope for PAb1620 on human p53 may correlate with wild type p53 suppressor function in a manner similar to the PAb246 epitope on murine p53. It was therefore of interest to determine the immunoreactivity of human p53 translated by the various rabbit reticulocyte lysates. Human p53 translated by lysates type A and group 1 was reactive with PAb1620 (Table II; Figure 2a). With lysates type B and group 2 the translated human p53 was negative for the PAb1620 epitope (Table II, Figure 2b). Reactivity with the monoclonal antibody PAb240 was similar to that observed for murine p53 in that murine p53-246$^o$ and human p53-1620$^o$ were positive for PAb240 and *vice versa* (Table II, Figure 2). Thus reactivity with PAb246/PAb1620 and PAb240 appears to be reciprocal on the native p53 protein.

We next compared the translation properties of reticulocytes lysate types A and B using murine p53 mRNA.

### *Kinetics of p53 mRNA translation*

The rate of p53 translation into $^{35}$S-labelled p53 was determined both by pulse-labelling and by continuous labelling with $^{35}$S-labelled methionine. Both methods gave essentially the same results; those for continuous labelling are shown in Figure 3. Maximal rates of $^{35}$S-methionine incorporation into p53 occurred within the first 30 min of translation and lysates type A and type B gave remarkably similar results (Figure 3).

The rate of appearance of conformation-dependent epitopes on the translated p53 protein was also investigated, taking aliquots of the translation mix at 10 min intervals for up to 90 min. With lysate type A the p53 was reactive with PAb246 from 10 min onwards; with lysate type B the PAb246 epitope was undetectable at all times (not shown). These results indicated that with lysate type B the p53 protein did not pass through a PAb246-positive form during the period of translation.

**Table I** Immunological reactivity of p53 translated *in vitro* and *in vivo*

| | Monoclonal antibody | | | | | | | | | |
|---|---|---|---|---|---|---|---|---|---|---|
| | *PAb421*[a] | *PAb122*[a] | *PAb248*[a] | *RA32C2*[a] | *PAb246*[b] | *PAb607*[b] | *PAb1620*[b] | *PAb200.47*[a] | *PAb242*[b] | *B5* |
| A | + | + | + | + | + | + | + | + | + | o |
| SV3T3 | + | + | + | + | + | + | + | + | + | o |
| B | + | + | + | + | o | o | o | + | + | o |
| 3T12 | + | + | + | + | o | o | o | + | + | o |

a and b: anti-p53 monoclonal antibodies directed against denaturation-stable epitopes (a) and denaturation sensitive epitopes (b). n.b. These monoclonal antibodies recognise seven distinct domains on the p53 protein.
A and B: p53 translated in reticulocyte lysate types A and B; SV3T3 and 3T12 = p53 from SV3T3 and 3T12 cell lysates. SV3T3 and 3T12 cells express wild type and mutant p53 respectively.

**Table II** Immunological reactivity of murine and human p53 translated *in vitro* using reticulocyte lysates prepared from individual rabbits

| | *PAb421* | *PAb248* | *RA3 2C2* | *PAb246* | *PAb1620* | *PAb240* | *PAb242* | *B5* |
|---|---|---|---|---|---|---|---|---|
| Group 1 | | | | | | | | |
| Murine p53 | + | + | + | + | + | o | + | o |
| Human p53 | + | NR | NR | NR | + | o | NR | o |
| Group 2 | | | | | | | | |
| Murine p53 | + | + | + | o | o | + | + | o |
| Human p53 | + | NR | NR | NR | o | + | NR | o |

NR = not reactive due to species specificity of the monoclonal antibody (Yewdell *et al.*, 1986).

**Figure 2** Immunoreactivity of human p53 translated by reticulocyte lysates prepared from individual rabbits. Examples of group 1 and group 2 lysates are shown, panels **a** and **b** respectively. Immunoprecipitations with PAb421 (lane 1), PAb1620 (lane 2) and PAb240 (lane 3). Exposure of autoradiographs: 24 h for panel a and 16 h for panel b.

**Figure 3** Kinetics of translation of p53 mRNA by reticulocyte lysates types A and B. Incorporation of $^{35}$S-methionine into TCA-precipitable counts at varying times of continuous labelling. Similar kinetics were obtained with pulse labelling over the same period. n.b. the incorporation of $^{35}$S-label into full length p53 protein was greater 95% TCA precipitable counts. X—X lysate type A; O—O lysate type B.

*Lysate mixing experiments*

The effect of lysate type B on p53-246$^{+}$, and of type A on p53-246$^{o}$, was tested by mixing and incubating the translated p53 protein with the appropriate lysate. The mixtures were incubated in the presence of anisomycin to block further protein synthesis. The effects of time, temperature and lysate ratios were all tested. Both p53-246$^{+}$ and p53-246$^{o}$ were stable in terms of their immunology and were not interconvertible by mixing respectively with lysates types B and A under any of the conditions tested (not shown).

Having demonstrated the stability of p53-246$^{+}$ and p53-246$^{o}$, once translated, we next tested the effect of translating p53 mRNA in mixtures of lysate type A plus type B. The results (Figure 4) indicated that lysate type B was dominant since, in the presence of 20% lysate type B, the level of p53-246$^{+}$ was reduced by 50%. In the presence of 50% lysate type B the p53 mRNA was translated into p53-246$^{o}$.

Overall, these results indicate that the presence/absence of the PAb246 epitope on wild type p53 is determined during the translation of the p53 polypeptide. This co-translational effect appears to be dependent upon factor(s) in lysate type B that suppresses the p53-246$^{+}$ conformation of p53.

*Phosphorylation of p53 translated in vitro*

The p53 protein *in vivo* may be phosphorylated at several sites, including serines 37, 312 and 389 (Samad *et al.*, 1986; Meek & Eckhart, 1987). Since the addition of one or more

**Figure 4** The immunology of p53 translated by mixtures of reticulocyte lysates type A and type B. Varying proportions of lysate types A and B were pre-mixed as indicated and p53 mRNA (1 μl per 10 μl mixed lysate) was added and translated in the presence of $^{35}$S-methionine at 30°C for 1 h. The translated product was immunoprecipitated and run out on SDS-PAGE (Materials and methods) and the amounts of $^{35}$S-labelled p53 immunoprecipitated with PAb246 relative to PAb421 were quantitated by scintillation counting (1 μl aliquots of immunoprecipitated p53) and by densitometry scanning of the autoradiographed gel. Dotted line indicates the level of background with B5, a negative control immunoprecipitation.

charged phosphate groups might affect the conformation of the p53 polypeptide we next tested the ability of reticulocyte lysate to phosphorylate p53 translated *in vitro*. The protein kinase activity of reticulocytes includes a component that is dependent upon cyclic AMP (cAMP) and accordingly the phosphorylation studies were carried out in the presence and absence of cAMP. Controls showed no effect of cAMP upon the conformation of p53 translated *in vitro*. The endogenous protein kinase activity of the reticulocyte lysate was high and multiple $^{32}$P-labelled proteins were detected after incubation with gamma $^{32}$P-ATP (not shown). Following immunoprecipitation with anti-p53 monoclonal antibodies, $^{32}$P-labelled p53 was clearly detectable (Figure 5a). Lysates type A and type B both phosphorylated p53 *in vitro*.

The wild type p53 variants were indistinguishable by two-dimensional gel electrophoresis (Figure 5b) and by peptide mapping (not shown), consistent with p53-246$^+$ and p53-246° representing conformational variants of the full length p53 polypeptide. Full length p53 is also indicated by reactivity with monoclonal antibodies directed against epitopes that span the wild type p53 polypeptide (Table I and Figure 1).

**Discussion**

The functioning of p53 involves molecular interaction with specific target proteins. The first cellular target to be identified for wild type p53 is p34$^{cdc2}$, a cell cycle control protein (Milner *et al.*, submitted).

The interaction between p53 and its target molecules will be determined in part by the conformation of the p53 polypeptide. For example p53-246$^+$ has a high affinity for the large T antigen of simian virus 40 (SV40), whereas mutant p53-246° fails to bind SV40 large T (Sturzbecher *et al.*, 1987). The wild type variant p53-246° also fails to bind SV40 large T (Milner & Cook, in preparation). Thus the presence/absence of the PAb246 epitope signifies two different functional states of the p53 protein. There is now good evidence that p53-246$^+$ (wild type) functions as a suppressor for cell proliferation, while p53-246° (mutant) has oncogenic properties (Hinds *et al.*, 1989; Baker *et al.*, 1989; Takahashi *et al.*, 1989).

We now demonstrate that wild type murine p53 can be expressed in the form p53-246°. This observation was completely unexpected since, hitherto, p53-246° was believed to represent mutant p53. The results presented in this paper are for wild type p53 expressed *in vitro*: the same stock of p53 mRNA being translated into p53-246$^+$ by lysate A and into p53-246° by lysate B (Figure 1, Table I). These results indicate that wild type p53 can exist in two specific tertiary conformations. This raises the possibility that wild type p53 is subject to allosteric control. Given the dominant function of wild type p53 as a suppressor for cell proliferation it is conceivable that the normal cellular response to growth stimulation will require transient inactivation of this p53 suppressor function. One mechanism could involve an allosteric change in p53, that is p53-246$^+$ to p53-246°. This would inactivate, for a transient period, p53 suppressor function and allow the cellular growth response. Indeed we now have good evidence that growth stimulation induces a conformational effect on wild type p53 *in vivo*, detected by loss of the PAb246 epitope (Milner & Watson, submitted).

Mutant p53-246° is associated with loss of p53 suppressor function. We now present evidence that wild type p53 can also adopt the p53-246° conformation. We propose that the two conformational states of wild type p53 may represent allosteric forms of p53, with negative (p53-246$^+$) and positive (p53-246°) functions in cell growth control. Mutation within the p53 gene may perturb tertiary folding of the p53 polypeptide in such a way as to destabilise p53 conformation associated with negative growth control.

**Figure 5** **a,** Immunoprecipitations of p53 translated *in vitro* in the presence of gamma $^{32}$P-ATP for 1 h at 30°C. Immunoprecipitations as follows: lane 1, PAb421; lane 2, RA3.2C2; lane 3, PAb246; lane 4, B5, a negative control. The results shown are for p53 mRNA translated with reitculocyte lysate type A. The position of p53 (arrowed) was determined using $^{35}$S-labelled p53 as marker (not shown). **b,** Two-dimensional gel electrophoresis of p53-246$^+$ and p53-246° translated *in vitro*. Reticulocyte lysate types A and B were used to translate p53 mRNA. The immunology of p53-246$^+$ (lysate type A) and p53-246° (lysate B) was confirmed by immunoprecipitations with PAb241 and PAb246 (not shown). Aliquots of the undiluted translation mix were subjected to two dimensional electrophoresis and autoradiography. Panel 1, 2 $\mu$l lysate type A (p52-246$^+$); panel 2, 2 $\mu$l lysate type B (p53-246°); panel 3, 1 $\mu$l lysate type A plus 1 $\mu$l lysate type B.

The availability of the monoclonal antibody PAb1620 allowed us to extend the above observations to human p53. PAb246 is species specific and does not recognise human p53. However, PAb1620 reacts with both murine and human p53 (Milner *et al.*, 1987). The PAb1620 and PAb246 epitopes are topologically related on murine p53 and they appear to be coupled in terms of their presence/absence on protein (Milner *et al.*, 1987, see Tables I and II). By extrapolation we predict that, for human p53, the PAb1620 epitope indicates wild type suppressor function. Moreover, activating mutations within the human p53 gene may yield p53-1620°, analogous to p53-246° for activated mutants of the murine gene. Thus the epitope for PAb1620 has potential as marker for the wild type p53 anti-oncogene.

We thank John Jenkins for p53 cDNA, David Lane and Ed Harlow for monoclonal antibodies, Tim Hunt for batches of pooled rabbit reticulocyte lysate and Tony Hunter for reticulocyte lysates prepared from individual rabbits. We also thank Peter Jackson for running the two-dimensional gels. This work was supported by a grant from the Cancer Research Campaign to J.M.

**References**

BAKER, S.J., FEARON, E.R., NIGRO, J.M. & 9 others (1989). Chromosomal 17 deletions and p53 gene mutations in colorectal carcinomas. *Science*, **244**, 217.

COFFMAN, R.L. & WEISSMAN, I.L. (1981). A monoclonal antibody which recognises B cells and B cell precursors in mice. *J. Exp. Med.*, **153**, 269.

DELEO, A.B., JAY, G., APPELLA, E., DUBOIS, G.C., LAW, L.W. & OLD, L.J. (1979). Detection of a transformation-related antigen in chemically induced sarcomas and other transformed cells of the mouse. *Proc. Natl Acad. Sci. USA.*, **76**, 2420.

ELIYAHU, D., GOLDFINGER, N., PINHASI-KIMHI, O. & 5 others (1988). Meth A fibrosarcoma cells express two transforming mutant p53 species. *Oncogene*, **3**, 313.

GAMBLE, J. & MILNER, J. (1988). Evidence that immunological variants of p53 represent alternative protein conformations. *Virology*, **162**, 452.

GURNEY, E.G., HARRISON, R.O. & FENNO, J. (1980). Monoclonal antibodies against Simian virus 40 T antigens: evidence for distinct subclasses of large T antigen and for similarities among non-viral T antigens. *J. Virol.*, **34**, 752.

HARLOW, E., CRAWFORD, L.V., PIM, D.C. & WILLIAMSON, N.H. (1981). Monoclonal antibodies specific for Simian virus 40 tumor antigens. *J. Virol.*, **39**, 861.

HARRIS, N., BRILL, E., SHOHAT, O., PROKOCIMER, M., WOLF, D., ARAI, N. & ROTTER, V. (1986). Molecular basis for heterogeneity of the human p53 protein. *Mol. Cell. Biol.*, **6**, 4650.

HINDS, P., FINLAY, C. & LEVINE, A.J. (1989). Mutation is required to activate the p53 gene for cooperation with the ras oncogene and transformation. *J. Virol.*, **63**, 739.

HUNT, T. & JACKSON, R.J. (1974). The rabbit reticulocyte lysate as a system for studying mRNA. In *Modern Trends in Human Leukaemia*, Neth, R., Gallo, R.C., Spiegelman, S. & Stohlman, F. (eds) p. 300. J.F. Lehmanns Verlag: Munich.

JENKINS, J.R., RUDGE, K., REDMOND, S. & WADE-EVANS, A. (1984). Cloning and expression analysis of full length mouse cDNA sequences encoding the transformation associated protein p53. *Nucl. Acids Res.*, **12**, 5609.

KESSLER, S.W. (1975). Rapid isolation of antigens from cells with a staphlyococcal protein A-antibody absorbant. *J. Immunol.*, **115**, 1617.

LAEMMLI, U.K. (1970). Cleavage of structural proteins during the assembly of the head of bacteriophage T4. *Nature*, **227**, 680.

MEEK, D.W. & ECKHART, W. (1987). Phosphorylation of p53 in normal and Simian virus 40-transformed NIH3T3 cells. *Mol. Cell. Biol.*, **8**, 461.

MERCER, W.E., NELSON, D., DELEO, A.B., OLD, L.J. & BASERGA, R. (1982). Microinjection of monoclonal antibody to protein p53 inhibits serum-induced DNA synthesis in 3T3 cells. *Proc. Natl Acad. Sci. USA*, **79**, 6309.

MILNER, J. & COOK, A. (1986). The cellular tumour antigen p53: evidence for transformation-related, immunological variants of p53. *Virology*, **154**, 21.

MILNER, J., COOK, A. & SHELDON, M. (1987). A new anti-p53 monoclonal antibody, previously reported to be directed against the large T antigen of simian virus 40. *Oncogene*, **1**, 453

MILNER, J. & MILNER, S. (1981). SV40-53K antigen: a possible role for 53K in normal cells. *Virology*, **112**, 785.

PELHAM, H.R.B. & JACKSON, R.J. (1976). An efficient mRNA-dependent translation system from reticulocyte lysates. *Eur. J. Biochem.*, **67**, 247.

REICH, N.D. & LEVINE, A.J. (1984). Growth regulation of a cellular tumor antigen, p53, in non-transformed cells. *Nature*, **308**, 199.

ROTTER, V., WITTE, O.N., COFFMAN, R. & BALTIMORE, D. (1980). Abelson murine leukaemia virus-induced tumors elicit antibodies against a host cell protein, p53. *J. Virol.*, **36**, 547.

SAMAD, A., ANDERSON, C.W. & CARROLL, R.B. (1986). Mapping of phosphomonoester and apparent phosphodiester bonds of the oncogene product p53 from Simian virus 40-transformed 3T3 cells. *Proc. Natl Acad. Sci. USA*, **83**, 897.

SOUSSI, T., FROMENTEL, C.C., MECHAL, M., MAY, P. & KRESS, M. (1987). Cloning and characterisation of a cDNA from Xenopus laevis coding for a protein homologous to human and murine p53. *Oncogene*, **1**, 71.

STURZBECHER, H.-W., CHUMAKOV, P., WELCH, W.J. & JENKINS, J.R. (1987). Mutant p53 proteins bind hsp 72/73 cellular heat shock-related proteins in SV40-transformed monkey cells. *Oncogene*, **1**, 201.

TAKAHASHI, T., NAU, M.M., CHIBA, I. & 7 others (1989). p53: A frequent target for genetic abnormalities in lung cancer. *Science*, **246**, 491.

YEWDELL, J.W., GANNON, J.V. & LANE, D.P. (1986). Monoclonal antibody analysis of p53 expression in normal and transformed cells. *J. Virol.*, **59**, 444.